# The Complex Inflammatory and Nutritional Indices to Predict Prognostic Risk for Patients With Acute Coronary Syndrome Undergoing Percutaneous Coronary Intervention

**DOI:** 10.1002/iid3.70180

**Published:** 2025-03-24

**Authors:** Ge Song, Xinchen Wang, Chen Wei, Yuewen Qi, Yan Liu, Ying Zhang, Lixian Sun

**Affiliations:** ^1^ Department of Cardiology The Affiliated Hospital of Chengde Medical University Chengde China; ^2^ Hebei Key Laboratory of Panvascular Diseases Chengde China; ^3^ Central Laboratory of Chengde Medical University Affiliated Hospital Chengde Hebei China; ^4^ The Cardiovascular Research Institute of Chengde Chengde China

**Keywords:** acute coronary syndrome, percutaneous coronary intervention, prognosis, systemic inflammation response index

## Abstract

**Purpose:**

To investigate the role of the systemic inflammatory response index (SIRI) and high‐density lipoprotein cholesterol (HDL‐C) and low‐density lipoprotein cholesterol (LDL‐C) levels in predicting the risk of major adverse cardiovascular events (MACEs) in patients with acute coronary syndrome (ACS) undergoing percutaneous coronary intervention (PCI).

**Patients and Methods:**

Overall, 1377 patients with ACS who underwent PCI between January 2016 and December 2018 were consecutively enrolled. The patients were divided into MACEs (*n* = 60) and non‐MACEs (*n* = 1317) groups. The study endpoints were MACEs, including cardiac‐related mortality and rehospitalization for severe heart failure (HF), myocardial infarction (MI), and in‐stent restenosis.

**Results:**

Both groups showed significant differences in the patients with age > 65 years, history of HF, acute MI, cardiogenic shock, left ventricular ejection fraction < 40%, SIRI ≥ 2.848, SIRI/HDL‐C ≥ 1.977, and SIRI × LDL‐C ≥ 4.609. The Kaplan–Meier curve showed that the low SIRI group had higher cumulative survival than the high SIRI group. Additionally, the univariate and multivariate Cox proportional hazards model demonstrated that SIRI ≥ 2.848, SIRI/HDL‐C ≥ 1.977, and SIRI × LDL‐C ≥ 4.609 were independent risk factors for patients with ACS undergoing PCI. Restricted cubic spline models were generated to visualize the relationship between SIRI, SIRI/HDL‐C, and SIRI × LDL‐C and the prognostic risk.

**Conclusion:**

SIRI ≥ 2.848, SIRI/HDL‐C ≥ 1.977, and SIRI × LDL‐C ≥ 4.609 were all independent prognostic risk factors in patients with ACS undergoing PCI, which may be useful markers for assessment for long prognosis.

## 1 Introduction

Cardiovascular disease remains the leading cause of death worldwide, with nearly half of these deaths due to ischemic heart disease [1, 2]. Acute coronary syndrome (ACS) is defined by a sudden reduction in blood supply to the heart [3]. Myocardial revascularization via percutaneous coronary intervention (PCI) is the standard treatment for patients with ACS [4].

Accurate assessment of prognostic risk is widely recognized to be important in improving the survival of ACS [5]. In recent years, more research has focused on different kinds of inflammatory indices as a convenient and noninvasive measure to diagnose and assess prognostic risks for ACS [6, 7, 8].

According to several clinical studies, the systemic inflammatory response index (SIRI) is a novel inflammatory biomarker [9] linked to the advancement of cardiovascular illnesses. Increased SIRI is an independent prognostic risk factor for overall survival in some cancers, for example, cervical cancer, nasopharyngeal carcinoma (NPC), gastroesophageal adenocarcinoma, hepatocellular carcinoma, and pancreatic cancer [10, 11, 12, 13, 14]. Our team have worked on the prognostic risk of different inflammatory indexes in patients with ACS since few years before. The previous study using SIRI‐based nomograms also showed the diagnostic significance of SIRI [15].

However, few studies have investigated the relationship between SIRI and ACS. The ability of SIRI combined with high‐density lipoprotein cholesterol (HDL‐C) or low‐density lipoprotein cholesterol (LDL‐C) as a marker to predict prognostic risk in patients with ACS undergoing PCI remains unknown. We, therefore, aimed to investigate the value of SIRI, the SIRI/HDL‐C ratio, and the SIRI × LDL‐C ratio in predicting the risk of major adverse cardiovascular events (MACEs) in patients with ACS undergoing PCI.

## 2 Patients and Methods

### 2.1 Study Design and Population

In this study, 1377 patients with ACS who underwent PCI between January 2016 and December 2018 at the Affiliated Hospital of Chengde Medical University were consecutively enrolled. All patients were treated according to international ACS practice guidelines [16]. The inclusion and exclusion criteria were the same as in our previous study [17]. Patient data during hospitalization were collected by postgraduates who received professional training using standard procedures. The diagnostic criteria for hypertension, type 2 diabetes mellitus, dyslipidemia, and ischemic stroke were as per our previous study [18]. All data were collected before discharge after ACS diagnosis and before PCI.

This study was approved by the Ethics Committee of the Affiliated Hospital of Chengde Medical University (approval number: CYFYLL2015006) and was conducted according to the tenets of the Declaration of Helsinki. All the participants provided informed consent.

### 2.2 Follow‐Up and Endpoints

Follow‐up data were collected via a review of electronic medical records and/or clinic visits at 1, 3, 6, and 12 months, and annually thereafter. The primary study endpoints were MACEs, including cardiac‐related mortality, rehospitalization for severe heart failure (HF), myocardial infarction (MI), or in‐stent restenosis.

### 2.3 Laboratory Data

Fasting blood samples were collected within the first 24 h of admission before PCI. White blood cell, platelet, neutrophil, and lymphocyte counts were assessed using an automatic hematology analyzer (Sysmex XE‐2100; Sysmex, Kobe, Japan). Hematologic inflammatory markers were calculated as follows:

SIRI=(neutrophils×monocytes)/lymphocytes,

SIRI/HDL‐C=[(neutrophils×monocytes)/lymphocytes]/high‐densitylipoproteincholesterol,

SIRI×LDL‐C=[(neutrophils×monocytes)/lymphocytes]×low‐densitylipoproteincholesterol.

### 2.4 Statistical Analysis

The sample size was calculated using software, PASS. The normality of the distribution of continuous variables was confirmed using the Kolmogorov–Smirnov test, and normally and nonnormally distributed variables were presented as the mean ± standard deviation and as the median with interquartile range, respectively. Differences in nonnormally distributed continuous variables between the MACEs and non‐MACEs groups were analyzed using the Mann–Whitney *U* test. Meanwhile, categorical variables were presented as numbers (%) and compared using the *χ* ^2^ test. Survival was estimated using the Kaplan–Meier method and compared between groups using the log‐rank test. The diagnostic value of SIRI, SIRI/HDL‐C, and SIRI × LDL‐C were evaluated using receiver operating characteristic (ROC) curves, and the optimal cutoff value was determined using Youden's index (sensitivity + specificity − 1). Significant variables in the univariate Cox proportional hazards model (i.e., those with *p* < 0.05) were entered into the multivariate Cox hazard proportional model. In the univariate and multivariate Cox hazard proportional hazards models, age was divided into two categories (< 65 and ≥ 65) as ranking variables. The R package rms was used to plot the restricted cubic spline (RCS). All statistical analyses were performed using SPSS (version 26; SPSS Inc., Chicago, IL), GraphPad Prism 8.0 (GraphPad Software Inc., La Jolla, CA), and R 4.3.3. *p* < 0.05 was considered statistically significant.

## 3 Results

### 3.1 Patient Characteristics

In total, 1377 patients who completed the follow‐up period were included in the final analysis. The median follow‐up time was 1146 days. Table 1 shows the characteristics of the patients in the MACEs (*n* = 60) and non‐MACEs (*n* = 1317) groups. The MACEs group and non‐MACEs group showed significant differences in the proportion of patients with age ≥ 65 years (22 [36.7%] vs. 319 [24.2%]), history of HF (19 [31.7%] vs. 122 [9.3%]), unstable angina (UA) (14 [23.3%] vs. 516 [39.2%]), acute myocardial infarction (AMI) (46 [76.7%] vs. 801 [60.8%]), cardiogenic shock (6 [10.0%] vs. 15 [1.1%]), left ventricular ejection fraction (LVEF) < 40% (7 [11.7%] vs. 34 [2.6%]), SIRI ≥ 2.848 (29 [48.3%] vs. 292 [22.2%]), SIRI/HDL‐C ≥ 1.977 (31 [51.7%] vs. 409 [31.1%]), SIRI × LDL‐C ≥ 4.609 (34 [56.7%] vs. 458 [34.8%]) (all *p* < 0.05) (Table 1). The MACEs group was related with age ≥ 65 years, history of HF, AMI, cardiogenic shock, LVEF < 40%, SIRI ≥ 2.848, SIRI/HDL‐C ≥ 1.977, and SIRI × LDL‐C ≥ 4.609.

**Table 1:** Baseline patient characteristics of the MACEs and non‐MACEs groups.

Variables	MACEs group (*n* = 60)	Non‐MACEs group (*n* = 1317)	*χ* ^2^/*Z*	*p* value
*Demographic*				
Male	42 (70.0%)	982 (74.6%)	0.627	0.429
Age ≥ 65 years	22 (36.7%)	319 (24.2%)	4.770	0.029
Dyslipidemia	34 (56.7%)	750 (56.9%)	0.002	0.996
Hypertension	33 (55.0%)	785 (59.6%)	0.505	0.477
Diabetes mellitus	16 (26.7%)	333 (25.3%)	0.058	0.810
Smoking	27 (45.0%)	682 (51.8%)	1.057	0.304
History of HF	19 (31.7%)	122 (9.3%)	31.337	< 0.001
Family history of CAD	5 (8.3%)	197 (15.0%)	2.012	0.156
UA	14 (23.3%)	516 (39.2%)	6.087	0.014
AMI	46 (76.7%)	801 (60.8%)	6.087	0.014
Cardiogenic shock	6 (10.0%)	15 (1.1%)	30.003	< 0.001
*Laboratory data*				
WBC (10^9^/L)	9.39 ± 3.31	8.68 ± 3.27	−1.858	0.063
Platelet (10^9^/L)	203.18 ± 54.02	220.18 ± 56.72	−1.794	0.073
Neutrophil count (10^9^/L)	6.40 (4.39, 9.40)	5.40 (3.95, 8.00)	−2.296	0.022
Lymphocyte count (10^9^/L)	1.39 (0.94, 1.96)	1.68 (1.24, 2.29)	−2.719	0.007
Monocyte count (10^9^/L)	0.48 (0.33, 0.75)	0.43 (0.32, 0.57)	−1.771	0.076
TC (mmol/L)	4.41 ± 1.12	4.43 ± 1.07	−0.025	0.980
TG (mmol/L)	1.42 (0.85, 2.27)	1.60 (1.03, 2.42)	−1.234	0.217
HDL‐C (mmol/L)	1.15 ± 0.34	1.11 ± 0.30	−0.606	0.544
LDL‐C (mmol/L)	2.41 ± 0.89	2.40 ± 0.84	−0.070	0.944
*Echocardiography*				
LVEDD > 50 mm	15 (25.0%)	330 (25.1%)	1.883	0.697
LVEF < 40%	7 (11.7%)	34 (2.6%)	16.396	< 0.001
*Coronary angiography*				
1 vessel	14 (23.3%)	418 (31.7%)	1.883	0.170
2 vessels	19 (31.7%)	418 (31.7%)	0.000	0.991
3 vessels	27 (45.0%)	481 (36.6%)	1.771	0.183
*Index*				
SIRI ≥ 2.848	29 (48.3%)	292 (22.2%)	21.970	< 0.001
SIRI/HDL‐C ≥ 1.977	31 (51.7%)	409 (31.1%)	11.212	0.001
SIRI × LDL‐C ≥ 4.609	34 (56.7%)	458 (34.8%)	12.837	< 0.001

*Note:* Data are presented as *n* (%) or as the median (range).

Abbreviations: AMI, acute myocardial infarction; CAD, coronary artery disease; HDL‐C, high‐density lipoprotein cholesterol; HF, heart failure; LDL‐C, low‐density lipoprotein cholesterol; LVEDD, left ventricular end‐diastolic diameter; LVEF, left ventricular ejection fraction; MACEs, major adverse cardiovascular events; SIRI, systemic inflammatory response index (neutrophil × monocyte‐to‐lymphocyte ratio); TC, total cholesterol; TG, triglyceride; UA, unstable angina; WBC, white blood cell.

### 3.2 Survival Analysis and ROC Curve

**Figure 1:**
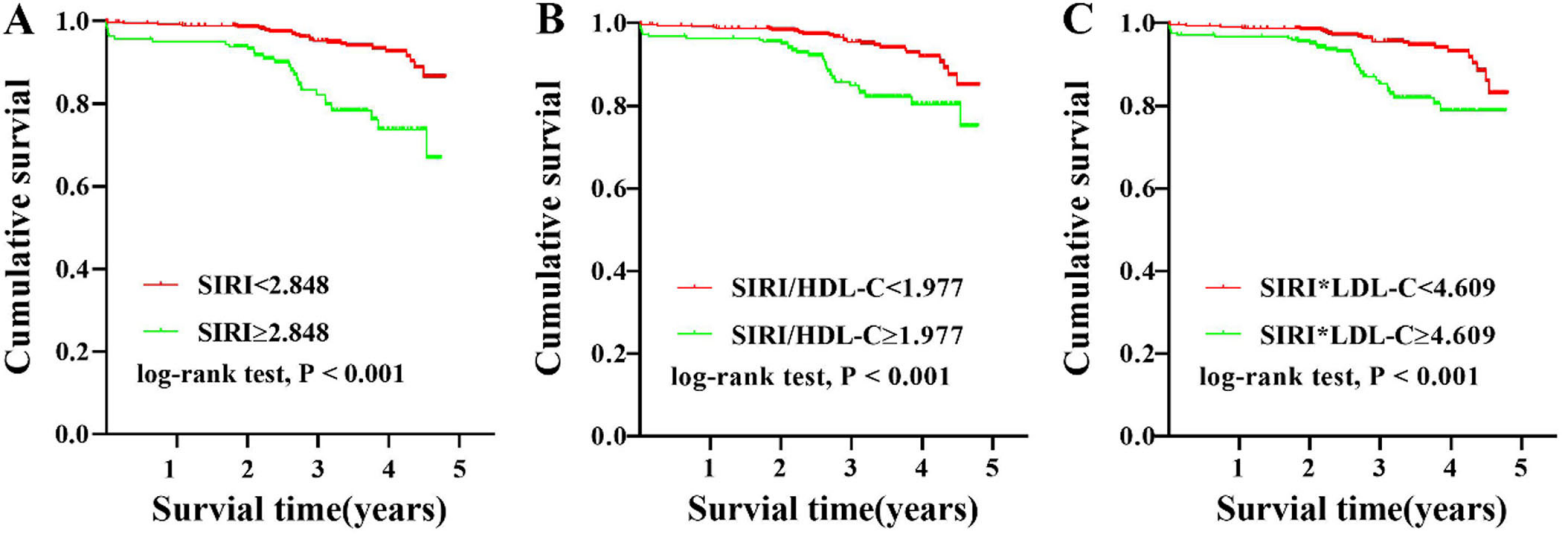
(A–C) Kaplan–Meier curves of cumulative survival by SIRI, SIRI/HDL‐C, and SIRI × LDL‐C in ACS patients undergoing PCI (log‐rank *p* < 0.001). ACS, acute coronary syndrome; HDL‐C, high‐density lipoprotein cholesterol; LDL‐C, low‐density lipoprotein cholesterol; PCI, percutaneous coronary intervention; SIRI, systemic inflammatory response index (neutrophil × monocyte‐to‐lymphocyte ratio).

**Figure 2:**
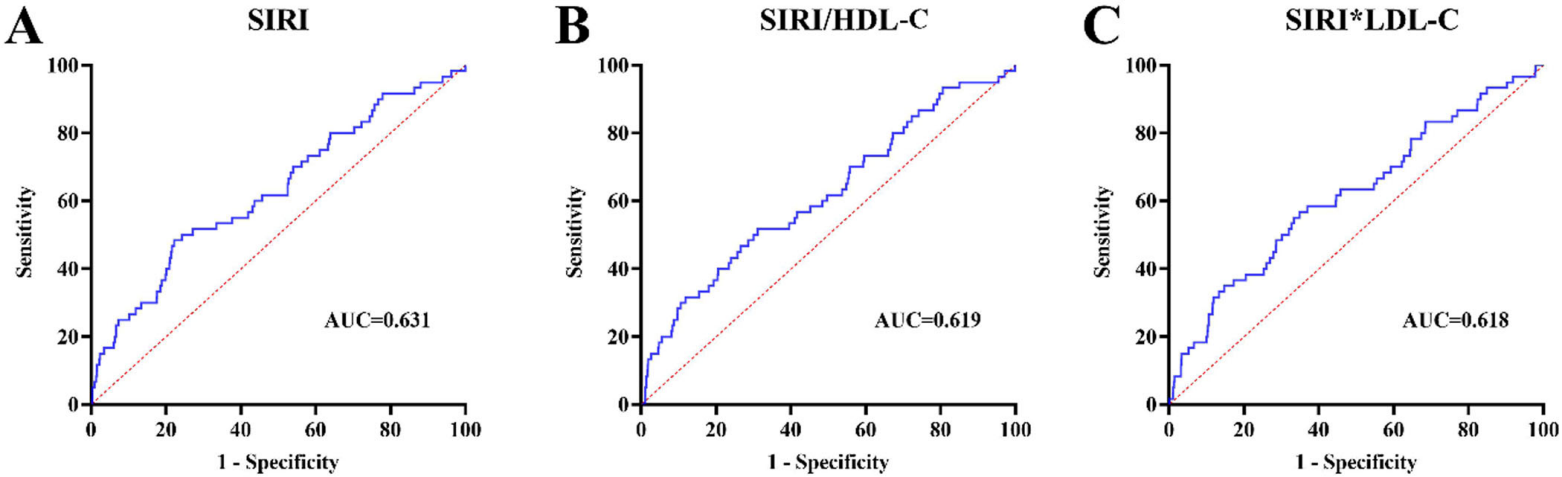
ROC curves. (A) Model 1 is adjusted for SIRI, (B) Model 2 is adjusted for SIRI/HDL‐C, and (C) Model 3 is adjusted for SIRI × LDL‐C. AUC, area under the curve; HDL‐C, high‐density lipoprotein cholesterol; LDL‐C, low‐density lipoprotein cholesterol; ROC curve, receiver operating characteristic curve; SIRI, systemic inflammatory response index (neutrophil × monocyte‐to‐lymphocyte ratio).

The Kaplan–Meier curve (Figure 1) showed that, compared with the group with SIRI ≥ 2.848, the group of SIRI < 2.848 had higher cumulative survival, and the difference was statistically significant (log‐rank *p* < 0.001). Similarly, high SIRI/HDL‐C and SIRI × LDL‐C levels also resulted in significantly lower cumulative survival (log‐rank *p* < 0.001). Table 2 presents the ROC curve analyses used to determine the optimal cutoff values for blood cell count‐derived inflammation indices for the evaluation of MACEs in patients with ACS undergoing PCI. The area under curve (AUC) for SIRI was 0.631 (*p* = 0.001, 95% confidence interval [CI]: 0.554–0.707) (Figure 2A). The optimal diagnostic cutoff point was 2.848, with a sensitivity of 48.3% and specificity of 78.8%. The AUC for SIRI/HDL‐C was 0.619 (*p* = 0.002, 95% CI 0.517–0.689) (Figure 2B), and the optimal diagnostic cutoff point was 1.977, with a sensitivity of 51.7% and specificity of 68.9%. The AUC for SIRI × LDL‐C was 0.618 (*p* = 0.002, 95% CI 0.541–0.695) (Figure 2C), and the optimal diagnostic cutoff point was 4.609, with a sensitivity of 56.7% and a specificity of 78.1%.

**Table 2:** ROC curve analyses of the blood cell count‐derived inflammatory indexes between the MACEs and non‐MACEs groups.

Variables	AUC (95% CI)	*p* value	Se (%)	Sp (%)	Cutoff
SIRI	0.631 (0.554–0.707)	0.001	48.3	78.8	2.848
SIRI/HDL‐C	0.619 (0.542–0.696)	0.002	51.7	68.9	1.977
SIRI × LDL‐C	0.618 (0.541–0.695)	0.002	56.7	78.1	4.609

*Note:* Data are presented as *n* (%) or as the median (range).

Abbreviations: AUC, area under the curve; CI, confidence interval; HDL‐C, high‐density lipoprotein cholesterol; LDL‐C, low‐density lipoprotein cholesterol; MACEs, major adverse cardiovascular events; ROC, receiver operating characteristic; Se, sensitivity; SIRI, systemic inflammatory response index (neutrophil × monocyte‐to‐lymphocyte ratio); Sp, specificity.

### 3.3 Univariate and Multivariate Cox Hazard Proportional Models

The univariate Cox proportional hazard model showed that a high SIRI (≥ 2.848) was an independent risk factor for patients with ACS undergoing PCI (hazard ratio [HR] 3.341, 95% CI 2.013–5.545, *p* < 0.001). A high SIRI/HDL‐C (≥ 1.977) and a high SIRI × LDL‐C (≥ 4.609) were also independent risk factors for patients with ACS undergoing PCI (HR 2.445, 95% CI 1.473–4.058, *p* = 0.001; HR 2.650, 95% CI 1.589–4.418, *p* < 0.001, respectively).

**Figure 3:**
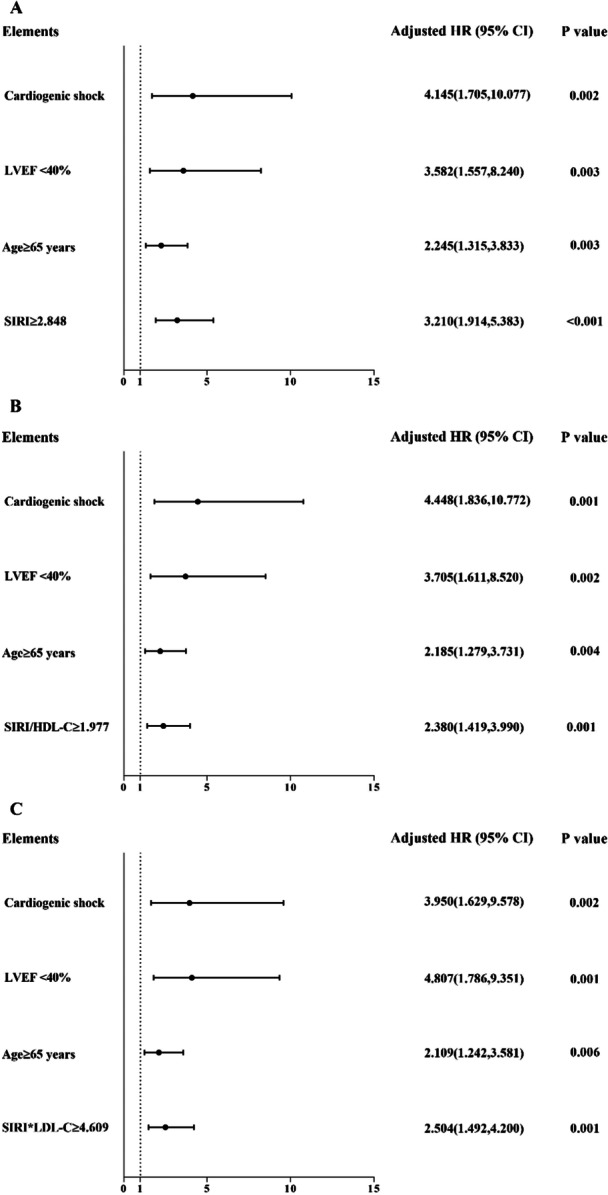
Forest graphs according to the Cox proportional hazards regression model to test the risk factors for MACEs. (A) Model 1 is adjusted for SIRI ≥ 2.848, age ≥ 65, cardiogenic shock, and LVEF < 40%; (B) Model 2 is adjusted for SIRI/HDL‐C ≥ 1.977, age ≥ 65, cardiogenic shock, and LVEF < 40%; (C) Model 3 is adjusted for SIRI × LDL‐C ≥ 4.609, age ≥ 65, cardiogenic shock, and LVEF < 40%. CI, confidence interval; HDL‐C, high‐density lipoprotein cholesterol; HR, hazard ratio; LDL‐C, low‐density lipoprotein cholesterol; LVEF, left ventricular ejection fraction; MACEs, adverse cardiovascular events; SIRI, systemic inflammatory response index (neutrophil × monocyte‐to‐lymphocyte ratio).

Other significant factors were age ≥ 65 years (*p* = 0.029), UA (*p* = 0.014), AMI (*p* = 0.014), history of HF (*p* < 0.001), and LVEF < 40% (*p* < 0.001). Age, cardiogenic shock, LVEF < 40%, and indices were finally selected in the multivariate Cox proportional hazards model through adjusted variables, and variables that effectively influenced the prognosis of patients with ACS were selected. After adjustment for age, cardiogenic shock, and LVEF < 40%, the multivariate Cox proportional hazard model showed that SIRI ≥ 2.848, SIRI/HDL‐C ≥ 1.977, and SIRI × LDL‐C ≥ 4.609 were independent risk factors for patients with ACS undergoing PCI: SIRI ≥ 2.848 (HR 3.210, 95% CI 1.914–5.383, *p* < 0.001), age ≥ 65 years (HR 2.245, 95% CI 1.315–3.833, *p* = 0.003), LVEF < 40% (HR 3.582, 95% CI 1.557–8.240, *p* = 0.003), cardiogenic shock (HR 4.145, 95% CI 1.705–10.077, *p* = 0.002) (Table 3 and Figure 3A); SIRI/HDL‐C ≥ 1.977 (HR 2.380, 95% CI 1.419–3.990, *p* = 0.001), age ≥ 65 years (HR 2.185, 95% CI 1.279–3.731, *p* = 0.004), LVEF < 40% (HR 3.705, 95% CI 1.611–8.520, *p* = 0.002), cardiogenic shock (HR 4.448, 95% CI 1.836–10.772, *p* = 0.001) (Table 3 and Figure 3B); SIRI × LDL‐C ≥ 4.609 (HR 2.504, 95% CI 1.492–4.200, *p* = 0.001), age ≥ 65 years (HR 2.109, 95% CI 1.242–3.581, *p* = 0.006), LVEF < 40% (HR 4.807, 95% CI 1.786–9.351, *p* = 0.001), cardiogenic shock (HR 3.950, 95% CI 1.629–9.578, *p* = 0.002) (Table 3 and Figure 3C).

**Table 3:** Cox hazard proportional models of MACEs risk according to blood cell count‐derived inflammatory indexes.

Indexes	Model 1		Model 2	
	HR (95% CI)	*p* value	HR (95% CI)	*p* value
SIRI	3.341 (2.013–5.545)	< 0.001	4.145 (1.705–10.077)	0.002
SIRI/HDL‐C	2.445 (1.473–4.058)	0.001	2.380 (1.419–3.990)	0.001
SIRI × LDL‐C	2.650 (1.589–4.418)	< 0.001	2.504 (1.492–4.200)	0.001

*Note:* Model 1, unadjusted; Model 2, adjusted for age category, cardiogenic shock, and LVEF.

Abbreviations: ACS, acute coronary syndrome; CI, confidence interval; HDL‐C, high‐density lipoprotein cholesterol; HR, hazard ratio; LDL‐C, low‐density lipoprotein cholesterol; LVEF, left ventricular ejection fraction; MACEs, major adverse cardiovascular events; PCI, percutaneous coronary intervention; SIRI, systemic inflammatory response index (neutrophil × monocyte‐to‐lymphocyte ratio).

### 3.4 Restricted Cubic Spline

**Figure 4:**
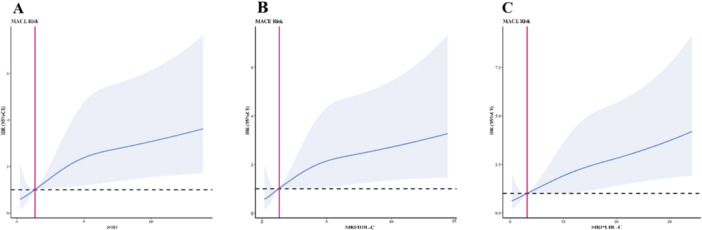
RCS models. (A) Model 1 is for SIRI, (B) Model 2 is for SIRI/HDL‐C, and (C) Model 3 is for SIRI × LDL‐C. CI, confidence interval; HDL‐C, high‐density lipoprotein cholesterol; HR, hazard ratio; LDL‐C, low‐density lipoprotein cholesterol; MACEs, major adverse cardiovascular events; RCS, Restricted cubic spline; SIRI, systemic inflammatory response index (neutrophil × monocyte‐to‐lymphocyte ratio).

RCS models were generated to visualize the relationships between SIRI, SIRI/HDL‐C ratio, SIRI × LDL‐C ratio, and prognostic risk. Patients with a high SIRI had more adverse events [19, 20] using RCS analysis. Model 1 (Figure 4A) was used for SIRI, Model 2 (Figure 4B) for SIRI/HDL‐C, and Model 3 (Figure 4C) for SIRI × LDL‐C. The risk of developing ACS in patients undergoing PCI increased with advanced SIRI, SIRI/HDL‐C, and SIRI × LDL‐C levels. As shown, when SIRI ≥ 1.338, SIRI/HDL‐C ≥ 1.271, and SIRI × LDL‐C ≥ 3.222, high SIRI, high SIRI/HDL‐C, and high SIRI × LDL‐C were independent risk factors.

## 4 Discussion

The main findings of this study are as follows. First, SIRI ≥ 2.848, SIRI/HDL‐C ≥ 1.977, and SIRI × LDL‐C ≥ 4.609 correlated with poor prognosis and were independent risk factors for ACS patients undergoing PCI. Patients with ACS who underwent PCI had high SIRI, high SIRI/HDL‐C, and high SIRI × LDL‐C values and a lower cumulative survival rate than those in the control group. The SIRI, SIRI/HDL‐C ratio, and SIRI × LDL‐C ratio were better predictors of MACEs in patients with ACS who underwent PCI. Second, the value of the novel indexes, SIRI, SIRI/HDL‐C, and SIRI × LDL‐C, had a similar ability to predict prognosis in patients with ACS who underwent PCI as classical indicators like age ≥ 65 years, cardiogenic shock, and LVEF < 40%. Finally, the HR trend of patients with ACS undergoing PCI was linear and increased gradually with SIRI, SIRI/HDL‐C, and SIRI × LDL‐C values. To the best of our knowledge, this is the first study to analyze the correlation between this novel index, SIRI with HDL‐C or LDL‐C, and the prognosis of patients with ACS who underwent PCI.

The SIRI was first developed in 2016, based on peripheral neutrophil, monocyte, and lymphocyte counts, to evaluate the ability to predict the survival of patients with pancreatic cancer [14]. Lipoprotein(a) is a risk factor for cardiovascular events, especially HDL‐C and LDL‐C [19]. HDL‐C quality features are associated with atherosclerotic disease [20]. LDL‐C levels after ACS are associated with cardiovascular risk reduction [21]. By combining the inflammatory indicator SIRI with HDL‐C and LDL‐C, a previous study derived the SIRI/HDL‐C and SIRI × LDL‐C indexes and proved that SIRI/HDL‐C and SIRI × LDL‐C had higher diagnostic efficiency for coronary heart disease (CHD). SIRI, SIRI/HDL‐C, and SIRI × LDL‐C levels are significantly correlated with the severity of coronary artery stenosis, which enhances the diagnostic accuracy of CHD [22]. However, the relationship between SIRI, SIRI/HDL‐C, and SIRI × LDL‐C and the prognosis of ACS remains unknown.

SIRI levels show the risk of cardiovascular mortality and are linked to the development and progression of various cardiovascular and respiratory conditions, including acute ischemic stroke, hypertension, HF, and idiopathic pulmonary arterial hypertension (IPAH). Furthermore, SIRI is widely used to forecast the outcome of cancer and other diseases, such as pneumonia. An increasing number of studies indicate that inflammatory markers play critical roles in the prognosis of various malignant tumors. The application of inflammatory indices in predicting the clinical outcomes of patients with stage IIB cervical cancer deserves popularization [10]. The overall survival of patients with higher SIRI values is significantly inferior to that of patients with lower SIRI values to predict the prognosis of NPC [11]. The SIRI has also shown robust prognostic value in patients with locally advanced gastroesophageal adenocarcinoma [12]. Inflammatory biomarkers such as platelet–lymphocyte ratio (PLR) and SIRI were significantly related to long‐term prognosis after liver transplantation for hepatocellular carcinoma. High SIRI and PLR were independent risk factors for poor prognosis, particularly SIRI, which was clearly associated with both overall and disease‐free survival [13]. Similarly, SIRI can be used to predict the survival of patients with pancreatic adenocarcinoma after gemcitabine‐based chemotherapy [14]. Furthermore, a previous study found that SIRI is an easily available and cost‐effective indicator that can be a potential aid in predicting the incidence of stroke‐associated pneumonia [23].

The SIRI has a strong relationship with cardiovascular disease [24, 25], and is closely related to the prognosis and diagnosis of acute ischemic stroke. Higher SIRI values indicate greater disease severity at admission and an increased incidence of poor outcomes at 90‐day prognosis [26]. The index has a better ability in predicting poor functional outcomes at discharge in acute ischemic stroke patients than the neutrophil–lymphocyte ratio, PLR, and lymphocyte‐to‐monocyte ratio [27]. Conversely, SIRI has been shown to be associated with AMI in previous studies [28].

A study performed a retrospective analysis of data from 1550 elderly patients with AMI, constructing a nomogram combining SII, SIRI, and partial medical history data (age, body mass index, previous stroke, and diabetes) at admission. The results showed that SIRI has a good predictive effect on the risk of in‐hospital death in elderly patients with AMI [28]. In 2024, a study investigated that, in patients with ischemic HF (IHF) undergoing PCI, increased SIRI was a risk factor for MACEs independent of other factors [29]. SIRI may represent a novel, promising, low‐grade inflammatory marker for the prognosis of patients with IHF undergoing PCI. In addition, SIRI is closely associated with high blood pressure, and plays a crucial role in IPAH, independently predicting its severity and prognosis. It is associated with various indicators of IPAH severity and is a significant predictor of clinical deterioration, offering additional predictive value beyond existing risk assessment scores [30].

However, there are few studies focusing on patients with ACS undergoing PCI. We used several approaches to investigate the correlation between SIRI, SIRI/HDL‐C, and SIRI × LDL‐C values and prognostic risk. Previous studies have shown that SIRI plays an important role in many tumors [10, 11, 12, 13, 14] and diseases [23, 24, 25, 26, 27, 28, 29, 30], especially cardiovascular diseases. Interestingly, by combining SIRI with HDL‐C and LDL‐C, the novel biomarkers, SIRI/HDL‐C and SIRI × LDL‐C, are complex indexes combining blood cell type and lipid measurements, which provide a more comprehensive understanding of systemic inflammation and lipid condition [22]. Calculating SIRI/HDL‐C and SIRI × LDL‐C can be easily and quickly obtained, and these indexes may improve the prognosis of patients undergoing PCI.

Our study showed that SIRI, as a novel index to predict the risk of MACEs, had the same efficiency as other classical prognostic risk factors. In addition, age, cardiogenic shock, and LVEF < 40% were the main factors affecting prognosis. The multivariate Cox proportional hazards model also demonstrated that SIRI ≥ 2.848, SIRI/HDL‐C ≥ 1.977, and SIRI × LDL‐C ≥ 4.609 were independent risk factors for patients with ACS undergoing PCI. In addition, an RCS plot was used to analyze the correlations between SIRI, SIRI/HDL‐C, SIRI × LDL‐C, and MACEs. Interestingly, the RCS curve showed that the HR of patients with ACS undergoing PCI increased gradually with the SIRI, SIRI/HDL‐C, and SIRI × LDL‐C values.

Our study has some limitations. First, this was a single‐center retrospective study with a small sample size. Second, owing to the lack of continuous monitoring of blood tests in this study, admission SIRI, HDL‐C, and LDL‐C levels were evaluated at a single time point, and fluctuations in SIRI, HDL‐C, and LDL‐C levels were not considered. Finally, all enrolled participants were Chinese; therefore, the association between these inflammatory composite measures and long‐term functional outcomes may need to be researched in populations from other countries.

## 5 Conclusion

Higher SIRI, SIRI/HDL‐C, and SIRI × LDL‐C levels were independently associated with a higher risk of MACEs in patients with ACS undergoing PCI. The above indices of inflammatory and nutritional status may be novel biomarkers to assess the long‐term prognosis in patients with ACS undergoing PCI.

## Author Contributions

All the authors contributed to the preparation of the manuscript and approved the final version. All authors contributed equally to this work. Study design: Ge Song, Xinchen Wang, Chen Wei, and Lixian Sun. Acquisition of data: Ying Zhang. Data analysis and interpretation: Ge Song, Xinchen Wang, and Yuewen Qi. Manuscript drafting: Ge Song and Lixian Sun. Critical revision of the manuscript for important intellectual content: Ge Song and Lixian Sun. Statistical analysis: Ge Song, Yan Liu, and Lixian Sun. Supervision: Lixian Sun.

## Ethics Statement

Ethical approval to report this case was obtained from the Institutional Review Board of The Affiliated Hospital of Chengde Medical University (Approval Number CYFYLL2015006).

## Consent

Written informed consent was obtained from the patients.

## Conflicts of Interest

The authors declare no conflicts of interest.

## Data Availability

The raw data supporting the conclusions of this article will be made available by the authors without undue reservation.
